# Pregnancy Outcome in Women With APECED (APS-1): A Multicenter Study on 43 Females With 83 Pregnancies

**DOI:** 10.1210/clinem/dgab705

**Published:** 2021-09-27

**Authors:** Saila Laakso, Elina Holopainen, Corrado Betterle, Viivi Saari, Elinor Vogt, Monica M Schmitt, Karen K Winer, Maria Kareva, Chiara Sabbadin, Eystein S Husebye, Elizaveta Orlova, Michail S Lionakis, Outi Mäkitie

**Affiliations:** 1 Children’s Hospital and Pediatric Research Center, University of Helsinki and Helsinki University Hospital, Helsinki, Finland; 2 Research Program for Clinical and Molecular Metabolism, Faculty of Medicine, University of Helsinki, Helsinki, Finland; 3 Folkhälsan Research Center, Helsinki, Finland; 4 Department of Obstetrics and Gynecology, University of Helsinki and Helsinki University Hospital, Helsinki, Finland; 5 Endocrine Unit, Department of Medicine (DIMED), University of Padua, Padua,Italy; 6 Department of Clinical Science and K.G. Jebsen Center for Autoimmune Diseases, University of Bergen, Norway; 7 Department of Medicine, Haukeland University Hospital, Bergen, Norway; 8 Laboratory of Clinical Immunology and Microbiology, National Institute of Allergy & Infectious Diseases (NIAID), National Institutes of Health (NIH), Bethesda, Maryland, USA; 9 Eunice Kennedy Shriver National Institutes of Child Health and Human Development (NICHD), National Institutes of Health, Bethesda, MD,USA; 10 Endocrinology Research Centre, Moscow, Russia

**Keywords:** fertility, delivery, premature ovarian insufficiency, autoimmunity

## Abstract

**Context:**

Autoimmune polyendocrinopathy-candidiasis-ectodermal dystrophy (APECED; also known as autoimmune polyendocrine syndrome type 1) has a severe, unpredictable course. Autoimmunity and disease components may affect fertility and predispose to maternal and fetal complications, but pregnancy outcomes remain unknown.

**Objective:**

To assess fetal and maternal outcomes and course of clinical APECED manifestations during pregnancy in women with APECED.

**Design and Setting:**

A multicenter registry-based study including 5 national patient cohorts.

**Patients:**

321 females with APECED.

**Main Outcome Measure:**

Number of pregnancies, miscarriages, and deliveries.

**Results:**

Forty-three patients had altogether 83 pregnancies at median age of 27 years (range, 17–39). Sixty (72%) pregnancies led to a delivery, including 2 stillbirths (2.4%) and 5 (6.0%) preterm livebirths. Miscarriages, induced abortions, and ectopic pregnancies were observed in 14 (17%), 8 (10%), and 1 (1.2%) pregnancies, respectively. Ovum donation resulted in 5 (6.0%) pregnancies. High maternal age, premature ovarian insufficiency, primary adrenal insufficiency, or hypoparathyroidism did not associate with miscarriages. Women with livebirth had, on average, 4 APECED manifestations (range 0-10); 78% had hypoparathyroidism, and 36% had primary adrenal insufficiency. APECED manifestations remained mostly stable during pregnancy, but in 1 case, development of primary adrenal insufficiency led to adrenal crisis and stillbirth. Birth weights were normal in >80% and apart from 1 neonatal death of a preterm baby, no serious perinatal complications occurred.

**Conclusions:**

Outcome of pregnancy in women with APECED was generally favorable. However, APECED warrants careful maternal multidisciplinary follow-up from preconceptual care until puerperium.

Maternal autoimmune diseases may pose a risk for adverse pregnancy outcomes (1-3). Autoimmune polyendocrinopathy-candidiasis-ectodermal dystrophy (APECED; OMIM #240300), also known as autoimmune polyendocrine syndrome type 1, is a rare autosomal recessive autoimmune disease. Mutations in the autoimmune regulator (*AIRE*) gene (21q22.3) lead to impaired expression of tissue-specific proteins in medullary thymic epithelial cell, resulting in the escape of self-reactive T cells to the periphery (4) and impaired T regulatory cell function (5). These defects in central immunological tolerance translate into organ-specific autoimmune disease characterized by infiltration by self-reactive T cells and production of multiple anticytokine and organ-specific autoantibodies (6).

The diagnosis of APECED is defined by the presence of 2 classical triad manifestations among chronic mucocutaneous candidiasis (CMC), hypoparathyroidism (HP), and primary adrenal insufficiency (PAI) or by 1 of these in a patient whose sibling has APECED (7). Clinical presentations are highly variable, and more than 20 different clinical manifestations have been described. New manifestations may appear throughout life (7-10). Up to 70% of women with APECED develop premature ovarian insufficiency (POI), but the incidence varies considerably (9%-70%) in different cohorts (11,12). Since POI is often diagnosed shortly after menarche (median age: 15-18 years) (8-10,12), it severely compromises fertility. Risk for adrenal crises and hypocalcemic seizures, worsening of genital candidiasis, and new autoimmune manifestations also have potentially negative effects on fertility and pregnancy outcomes.

Despite these factors, data on fertility and pregnancy outcome in women with APECED are scarce, and there are no guidelines for preconceptual care or pregnancy management. A Russian study reported 9 successful and uncomplicated pregnancies in 7 women (9). An earlier Finnish report described both spontaneous and ovum donation (OD) pregnancies in 7 women with APECED (7). Alkhammas et al reported a single successful OD pregnancy in which intravenous immunoglobulin was used throughout the pregnancy to prevent maternal autoimmunity toward the fetus. The pregnancy was complicated by gestational diabetes and preeclampsia in gestational week 37 (13).

Collecting and reporting data on reproductive outcomes is crucial to guide patient care and to improve the understanding of the effects of autoimmunity on fertility and pregnancy outcomes in APECED. Moreover, the impact of pregnancy on APECED manifestations is unknown. Lack of knowledge may affect the patients’ family planning. To describe pregnancy outcomes and pregnancy-related changes in clinical manifestations in women with APECED, we collected data from 5 large national patient cohorts.

## Patients and Methods

### Patients

We collected the data retrospectively from Italian (IT), Finnish (FIN), Russian (RUS), American (USA), and Norwegian (NO) cohorts of patients with APECED. The recruitment of patients into the cohorts has been described previously (9,10,14-16). These national cohorts include in each country a substantial part or all patients with APECED. The study was approved by the Ethics Committee of the Azienda Ospedaliera-Universitaria of Padua, Italy (Ref. no 1299P and 1583P); the Research Ethics Committee of the Hospital District of Helsinki and Uusimaa; the Ethics Committee at Endocrinology Research Centre of Russia; and the Norwegian Regional Committee for Medical and Health Research Ethics (permit no. 2018/1776/REK Vest). Patients seen at the National Institutes of Health Clinical Center in the United States were enrolled in an institutional review board–approved prospective natural history study (NCT01386437). All patients or their parents or guardians signed the informed consent.

### Clinical Data

*AIRE* genotype, age at diagnosis of each APECED manifestation, details related to pregnancies (maternal age, infertility treatments, outcome of pregnancies, pregnancy-related diseases), fetal birth characteristics, and the clinical course of APECED during pregnancy, delivery, and puerperium were collected from the data obtained during clinical study visits, by patient interviews, and from medical records. The data on Finnish patients were supplemented with data from the national registries of Health and Welfare Institute and Finnish Pregnancy Termination Registry using Finnish personal identity codes as patient identifiers.

In all cohorts, age at diagnosis was collected for the following APECED manifestations: CMC, HP, PAI, diabetes mellitus, growth hormone deficiency, POI, alopecia, enamel dysplasia, gallbladder stones, hepatitis, keratitis, malabsorption, nephritis, pernicious anemia, and vitiligo. The diagnosis of POI was in the FIN, RUS, NO, and USA cohorts based on elevated plasma levels of follicle-stimulating hormone or luteinizing hormone, amenorrhea, and absent, slow, or regressing pubertal development (8-10,12). In the IT cohort, diagnostic criteria of POI included the same criteria as in other cohorts and, in addition, the presence of 21-hydroxylase autoantibodies and 1 or more of the following 3 autoantibodies: steroid-producing cells autoantibodies, side chain cleavage autoantibodies, or 17α-hydroxylase autoantibodies (16). Diagnoses of hypothyroidism were collected from FIN, RUS, NO, and USA cohorts, and diagnoses of Hashimoto’s thyroiditis were collected from the IT cohort. Hashimoto’s thyroiditis was defined by the presence of at least 2 of the 3 following criteria: (1) thyroid autoantibodies, (2) typical ultrasonographic pattern, and (3) hypothyroidism. Data on intestinal dysfunction were collected for the USA and NO cohorts, and data on urticarial eruption were collected for the FIN and USA cohorts.

### Birth Measurements

Delivery between gestational weeks 37 and 42 was considered full-term. We transformed birth weights and lengths to Z-scores according to Finnish growth references (17). Z-scores were calculated according to exact gestational age or if gestational age was marked as full-term, reference values at gestational week 40 + 0 were used.

### Statistics

Characteristics are presented with medians (range). Association between maternal age and risk of miscarriage was tested with logistic regression. Fisher’s exact test was used to test for the differences in the prevalence of HP, PAI, or POI between the pregnancies ending in a miscarriage or stillbirth in comparison to a livebirth. Statistical analyses were conducted with GraphPad Prism 8 for macOS (version 8.4.3).

## Results

### Cohort Characteristics

A total of 321 female subjects with APECED were identified in the IT, FIN, RUS, USA, and NO cohorts. The patients’ median age in the cohorts ranged from 21 to 46 years, and in the entire cohort, the age range was 3 to 77 years. A total of 240 subjects were older than 16 years, representing currently or previously potentially fertile proportion of the cohort. Cohort characteristics are presented in Table 1. Altogether, 43 women had been pregnant, and their median age at the time of data collection was 46 years (range: 22-71 years). The proportion of >16-year-old patients with a history of pregnancy varied from 13% to 26% in different cohorts.

**Table 1.** Characteristics of the 5 national cohorts of females with autoimmune polyendocrinopathy-candidiasis-ectodermal dystrophy

	Total female patients/female >16 years, n/n	Age at the end of follow-up, years		>16 year-old patients with pregnancy, n (%)	Genotype (pregnant women)	Number
Country		Mean	Median (range)			
Italy	100/82	32	31 (3-77)	13 (16)	*c.*769C>T/*c.*967_979del13	1
					*c.*769C>T/*c.*232T>A	1
					*c.*967_979del13/*c.*967_979del13	1
					Other^a^	6
					Unknown	4
Finland	49/47	40	41 (8-69)	12 (26)	*c.*769C>T/*c.*769C>T	9
					*c.*769C>T/*c.*932G>A	2
					*c.*769C>T/*c.*1163^1164insA	1
Russian	88/55	22	21 (4-49)	7 (13)	*c.*769C>T/*c.*769C>T	2
					*c.*769C>T/*c.*173C>T	1
					*c.*769C>T/*c.*661A>T	1
					*c.*769C>T/*c.*232T>A	1
					*c.*769C>T/unknown	1
					Unknown	1
United States	61/34	24	22 (3-68)	6 (18)	*c.*967_979del13/*c.*967_979del13	2
					*c.*967_979del13/*c.*1163_1164insA	2
					*c.*967_979del13/*c.*232T>C	1
					*c.*967_979del13/*c.*1616C>T	1
Norway	23/22	51	46 (9-69)	5 (23)	*c.*769C>T/*c.*769C>T	2
					*c.*967_979del13/*c.*879+1G>A	1
					*c.*967_979del13/*c.*1336C>G	1
					Other^a^	1

^a^Other variants that 7 patients carried without major mutations included: *c.*232T>A, p.(Trp78Arg); *c.*588C>T, p.(Ser196Ser); *c.*682T>G, p.(Gly228Trp); *c.*755C>T, p.(Pro252Leu); *c.*681C>T, p.(Gly277Gly); *c.*834C>G, p.(Ser278Arg); *c.*1197T>C, p.(Ala399Ala); and an intronic variant *c.*879+1G>A.

Biallelic *AIRE* mutations had been identified in 38 of the 43 subjects with pregnancies (Table 1). Twenty-two patients (51%) were homozygous or heterozygous for *c.*769C>T, p.(Arg257Ter), and 9 patients (21%) were homozygous or heterozygous for *c.*967_979del13, p.(Leu327fs). In 5 cases, APECED diagnosis was set according to clinical criteria.

### Pregnancies

Altogether, 83 singleton pregnancies were registered. Seventy-five (90%) were spontaneous while in 8 (10%) cases, pregnancies were the result of infertility treatments (ovulation induction or in vitro fertilization; n = 3) or ODs (n = 5). Sixty pregnancies (72%) led to delivery. Of these, 54 (90%) were full-term and 6 (10%) preterm, including 2 stillbirths in gestational weeks 34 and 37 (Table 2). One of them was related to APECED complicated by adrenal crisis. The other was an OD pregnancy complicated by hypertension and a uterus anomaly unrelated to APECED.

**Table 2.** Deliveries and offspring’s characteristics in 5 national autoimmune polyendocrinopathy-candidiasis-ectodermal dystrophy cohorts

Country	Deliveries	Full-term	Preterm	Spontaneous/infertility treatment/ovum donation	Birth weight, kg, median (range)	Weight within −2 SD ± 2 SD, y/n/NA	Birth length, cm, Median (range)	Length within −2 SD ± 2 SD, y n/NA
Italy	15	14	1	15/0/0	3.78 (2.80-5.00)	8/2/5	51.0 (48.5-55.0)	9/1/5
Finland	12	11	1	8/1/3	3.08 (2.48-3.33)	10/1/1	47.5 (45.0-51.0)	9/0/3
Russian	12	10	2	12/0/0	3.00 (0.48-4.50)	8/3/1	50.0 (23.0-56.0)	8/3/1
United States	10	8	2	10/0/0	2.46 (2.27-3.59)	7/1/2	48.3 (45.7-48.3)	3/0/7
Norway	11	11	0	8/2/1	3.62 (3.21-4.65)	10/1/0	50..5 (46.0-53.0)	9/1/1
Total	60	54	6	53/3/4	3.14 (0.48-5.00)	43/8/9	50.0 (23.0-56.0)	38/5/17

Abbreviation: NA, not applicable.

There were 14 miscarriages (17% of all pregnancies) in 11 women. All but 1 occurred in the first trimester. Seven of these women also had pregnancies ending in a livebirth. Two women had recurrent miscarriages, defined as loss of 2 consecutive pregnancies (18). No screening for acquired or hereditary thrombophilia was performed for the patients with recurrent miscarriages. Eight (10%) women had induced abortions, but none were induced because of fetal malformation. One ectopic pregnancy was reported.

**Figure 1.**
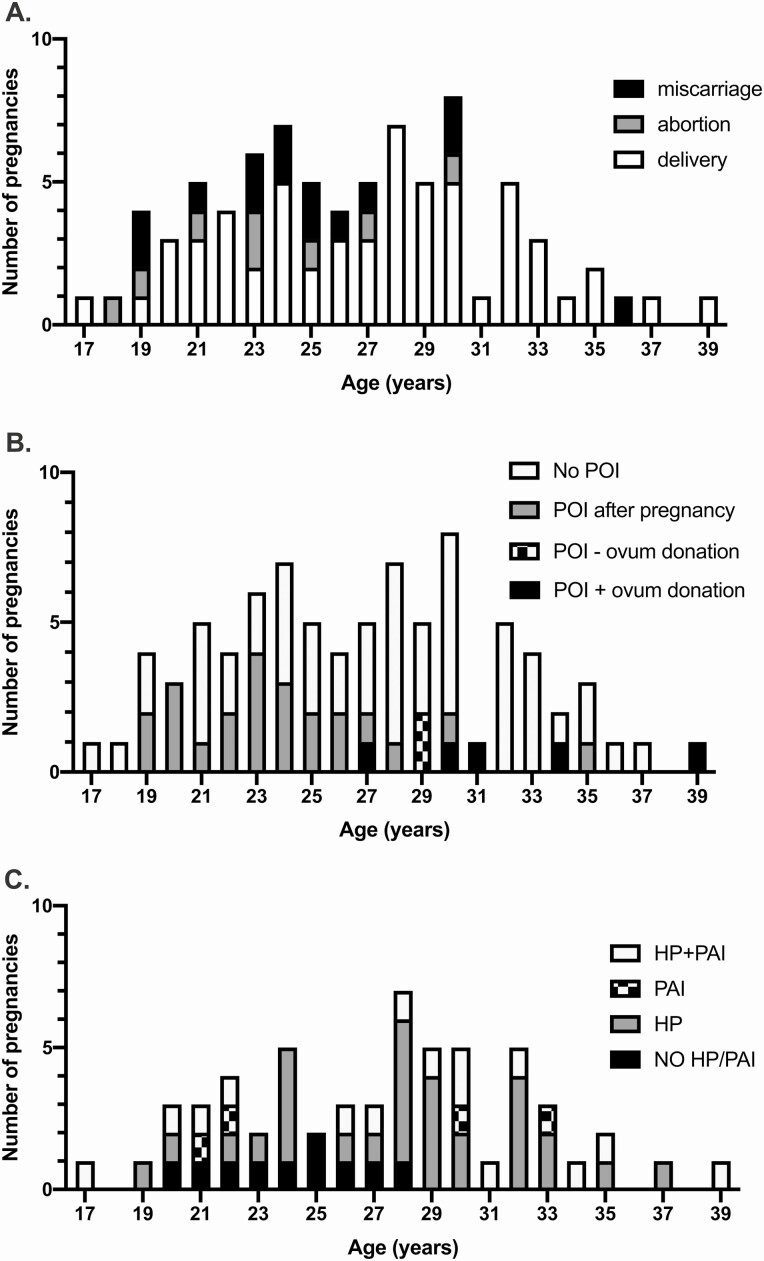
Pregnancies in women with autoimmune polyendocrinopathy-candidiasis-ectodermal dystrophy (APECED). (A) Pregnancy outcome according to age in women with APECED (age range: 17-39 years). (B) Pregnancies in patients according to history of premature ovarian insufficiency (POI). (C) Presence of hypoparathyroidism (HP) and primary adrenal insufficiency (PAI) in women with APECED in pregnancies leading to delivery of live-born baby according to maternal age.

Median age at the beginning of pregnancy was 27 years (range: 17-39 years) (Fig. 1A). In pregnancies ending in a miscarriage, the maternal age ranged from 19 to 36 years (median: 24 years) and, in induced abortions, from 18 to 30 years (median: 22 years). Higher maternal age did not predispose to miscarriage [maternal age in 16 miscarriages/stillbirths vs 58 livebirths; odds ratio (OR) 0.96, 95% CI 0.8-1.07, *P *= 0.35]. Detailed data on pregnancies leading to delivery in different subcohorts are presented in Table 2.

### Pregnancy History and Development of POI

Five patients were diagnosed with POI before the first pregnancy. Among them, there were 7 pregnancies, 5 of which were OD pregnancies. Two patients with POI became spontaneously pregnant and had a term delivery 3 and 10 years after POI diagnosis. The latter had a history of successful OD pregnancy 2 years before a spontaneous pregnancy. In both cases, POI diagnosis was based on amenorrhea and elevated follicle-stimulating hormone levels.

Pregnancies in patients with and without history of POI are presented in Figure 1B. Seventeen women were diagnosed with POI before the age of 40 (median: 28 years, range: 13-39 years). Eight women reached physiological menopause at the median age of 47 years (range: 43-53 years). In those who had either POI or physiological menopause, the time interval between spontaneous pregnancy and POI or menopause ranged from 2 to 34 years (median: 13 years). Seven pregnancies began less than 5 years before POI diagnosis, 4 of them leading to a delivery and 3 to spontaneous miscarriage. When we compared the pregnancies between subjects with (n = 27) and without POI (n = 47), the prevalence of miscarriage or stillbirth did not differ (OR 2.05, 95% CI 0.70-5.78, *P *= 0.25).

### Pregnancy Complications

Gestational hypertension was reported in 5 (5/60; 8%) pregnancies of which 1 ended in a preterm livebirth. Preeclampsia was diagnosed in 4 (4/60, 7%) pregnancies; 2 ended in preterm and 2 in full-term livebirths. Among the 5 OD pregnancies, no preeclampsia was diagnosed, and gestational hypertension was diagnosed in a pregnancy ending in stillbirth. One patient was diagnosed with gestational diabetes; none had hepatogestosis or chorioamnionitis. None of the patients were diagnosed with antiphospholipid syndrome. Placental complications were reported in 3 cases: 1 placental abruption, 1 partial placenta previa, and 1 placenta accreta.

### Offsprings’ Birth Characteristics

Birth weight was within normal range (Z-score −2.0 to +2.0) in the majority of children (43/51; 84%); 3 had birth weight Z-score above +2.0, and 5 below −2.0. Detailed birth measurements are presented in Table 2. A neonatal death was reported in a preterm baby (birth weight 480 g) who died at gestational week 26.

### APECED Manifestations and Pregnancy Outcome

**Figure 2.**
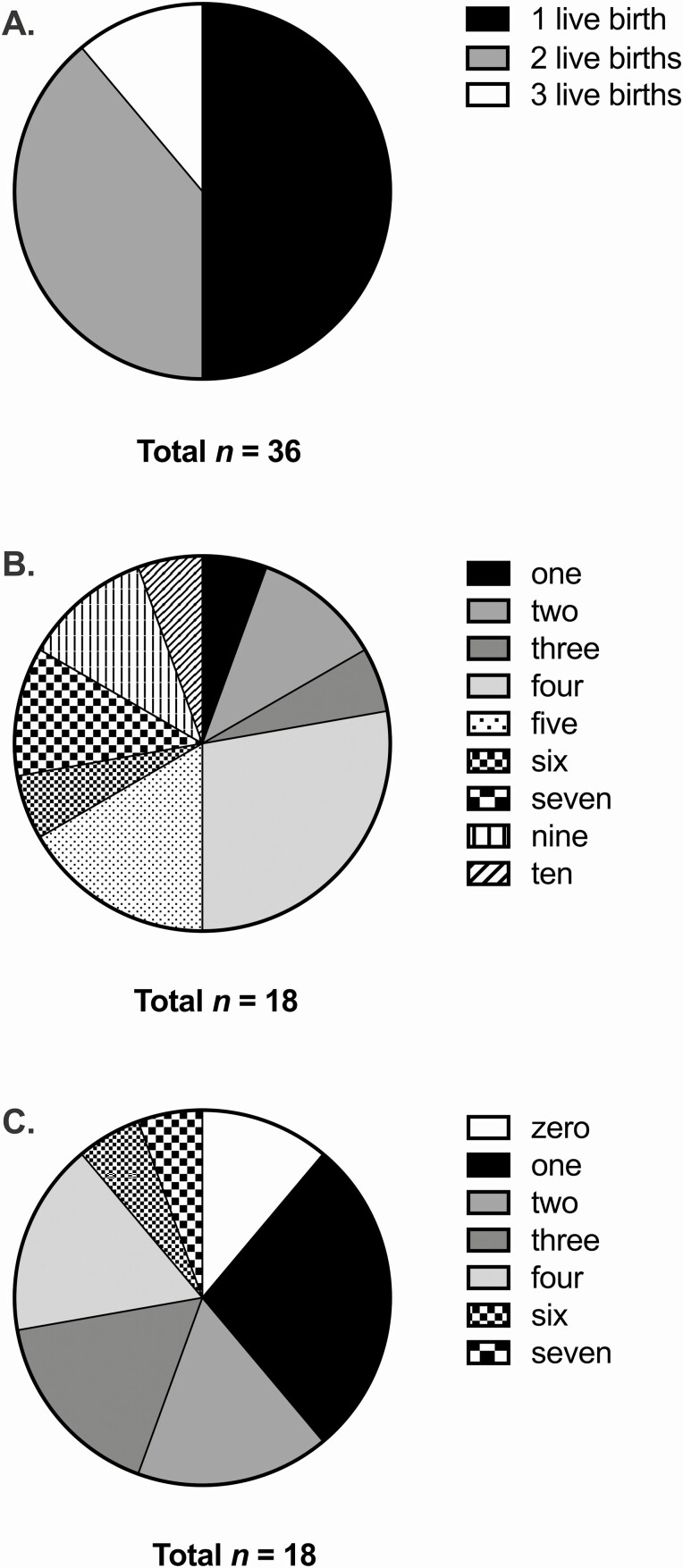
Livebirths in women with autoimmune polyendocrinopathy-candidiasis-ectodermal dystrophy (APECED). (A) Number of livebirths in 36 women with APECED. (B) Number of APECED manifestations at pregnancy in 18 patients with a livebirth. (C) Number of APECED manifestations at last pregnancy in 18 patients with 2 to 3 livebirths.

The APECED phenotypes varied greatly among the 43 pregnant women. The median age at the time of first disease manifestation was 4 years (range: 0.5-30 years). Three patients had 6 pregnancies before the development of any APECED manifestations between the ages of 18 and 26 years. Thirty-six women with at least 1 livebirth (36/240 of women >16 years old; 15%) presented a median of 4 disease manifestations in their first pregnancy (range: 0-10). Of these women, 78% had HP, 36% had PAI, and 31% had both HP and PAI (Fig. 1C). The distribution of the total number of disease manifestations varied according to number of livebirths (Fig. 2A-2C). Among 18 patients with 2 to 3 singleton livebirths, 72% had no more than 3 APECED manifestations. Three were diagnosed with a new manifestation between the first and later deliveries (Hashimoto’s thyroiditis, CMC, and PAI).

Four women had only pregnancies leading to miscarriage or ectopic pregnancy; 2 of them had at least 6 APECED manifestations. When we compared the pregnancies in subjects with or without PAI (n = 24 vs n = 50) and with or without HP (n = 58 vs n = 16), there were no differences in combined frequencies of miscarriages and stillbirths (patients with PAI: OR 0.93, 95% CI 0.32-3.06, *P *> 0.99; patients with HP: OR 0.78, 95% CI 0.24-2.54, *P *= 0.74). Four patients with a total of 5 preterm livebirths had 2 to 9 APECED manifestations. All had HP and 1 had PAI. One patient with HP had 2 preterm deliveries related to preeclampsia.

### Changes in APECED Manifestations During Pregnancy

The vast majority of patients felt well and their APECED manifestations were stable during pregnancy. Only 1 of the 43 patients developed a new APECED manifestation while pregnant. A 35-year-old patient developed PAI during her second spontaneous pregnancy, and an adrenal crisis led to intrauterine fetal death in gestational week 34. In addition, a 30-year-old patient with 9 disease manifestations needed long-term stress dosing of corticosteroids for PAI during the second trimester and was diagnosed with gestational diabetes in week 24. The pregnancy ended in preterm livebirth of a healthy child with normal birth weight in gestational week 36. Stress dosing of corticosteroids for PAI during deliveries was performed according to institutional recommendations, and no one developed adrenal crisis during delivery or caesarean section.

In patients with HP, 46 pregnancies (79%) ended in a livebirth. Four needed moderate modifications of medications due to hypocalcemia. One patient with HP developed hypercalcemia during the third trimester and hypocalcemia soon after delivery. In this patient, frequent dose adjustments of HP medication were needed during breastfeeding. None of the patients experienced severe hypocalcemia with seizures during pregnancies, deliveries, or puerperium.

In 1 patient, alopecia worsened during the 2 spontaneous pregnancies. No worsening of other disease manifestations, including CMC, was reported. Three patients had autoimmune hepatitis, and immunosuppressive medications were discontinued in all of them before pregnancies. A 28-year-old patient underwent kidney transplantation due to tubulointerstitial nephritis 8 years before a spontaneous pregnancy ending in full-term livebirth; she used immunosuppressive medications during pregnancy without any adverse effects.

## Discussion

Previous literature on fertility and pregnancy outcomes in APECED is scarce (7,9,13), and there are no guidelines regarding preconceptual care or clinical management of women with APECED during gestation. In this first pooled multicenter study of pregnancy course and outcomes in APECED, we found a low number of pregnant APECED women, which was expected given the high frequency of POI at early age. Incidence of miscarriages (17%) or preterm deliveries (10%) was not increased in comparison to the general population. The clinical spectrum of APECED disease manifestations remained mostly stable during pregnancy except for 1 case where PAI developed during pregnancy, resulting in adrenal crisis and fetal demise at gestational week 34. The children’s birth weights were usually normal, and apart from 1 neonatal death in a premature infant, the children presented no other serious manifestations during perinatal period.

A clear trend was that pregnant APECED women overall had a relatively mild APECED phenotype: half of the women whose pregnancies ended with a livebirth had less than 4 APECED manifestations, which is less than average in patients with APECED (7,9,10). More than 70% of women with 2 to 3 children had 3 or fewer manifestations. This trend is underpinned by the fact that 6 pregnancies in 3 women occurred before the first APECED manifestation appeared.

One of the most common endocrinopathies in women with APECED is an early-onset POI, negatively affecting fertility (11,12). In our series, 40% of women were diagnosed with POI at the median age of 28 years, either before or after pregnancies. The prevalence of POI was lower than reported in national cohorts (70%) (11). Autoimmune POI is a consequence of ovarian degeneration, caused by destruction of the growing theca cells with subsequent decrease in estrogen production and compensatory rise in circulating gonadotropins, but granulosa cells with quiescent follicles may remain functionally intact (19,20). Thus, in autoimmune POI the number and quality of ovums is not affected by genetic or chromosomal aberrations, which are typical in the normal aging ovary with diminished ovarian reserve. Multifactorial etiology for reduced fertility has been suggested. In addition to autoimmune oophoritis, delayed embryonic preimplantation development and compromised implantation may also play a role (21). Intermittent and unpredictable ovarian function and spontaneous ovulations can happen in autoimmune POI (22). In our cohorts, 5 patients were diagnosed with POI before the first pregnancy. They had a history of 5 OD pregnancies and 2 spontaneous pregnancies, reflecting intermittent ovarian function. However, spontaneous pregnancies are rare in POI (22), and no guidelines for the treatment of autoimmune POI are available. OD is the method of choice for infertility treatment in patients with POI, provided that other maternal factors do not pose risks for the pregnancy.

Autoimmunity, in general, may predispose to miscarriages (1). In 1 report on women with adrenal insufficiency, the percentage of miscarriages was 27.3% (15/55) (23). The miscarriage rate in our cohort was 17%, which is not increased compared with the general population (15%-25%) (24). In our study, higher maternal age, POI, PAI, or HP was not associated with increased risk for miscarriage. Recurrent miscarriages have been reported more often in women with several autoimmune disorders in comparison to women with only Hashimoto’s thyroiditis (25). Our study cohorts included 2 patients with recurrent miscarriages; the possibility of antiphospholipid syndrome was not investigated. Autoimmune diseases are also associated with preterm deliveries (2,3). In 2013, 11.4% of all babies were born preterm in the United States (26). In our cohort, 91.4% (53/58) of liveborn babies were born full-term, and there was only 1 early preterm birth (gestational age 26 weeks), giving a prematurity rate of 8.6%. Preeclampsia complicated 2 out of 5 pregnancies leading to preterm livebirth. Immunological mechanisms play a significant role in preeclampsia (27). Its incidence is generally higher in OD pregnancies (28), but we found none, nor did they end in premature birth. In the pregnancies leading to delivery, the prevalence of gestational hypertension or preeclampsia was not markedly increased in comparison to the worldwide prevalence (29).

In addition to preeclampsia, PAI may increase the risk for preterm delivery. In a recent report on pregnancy outcome in women with adrenal insufficiency, 25/117 (21.4%) deliveries were reported as preterm (23), which is significantly higher rate than 8.3% (2/24; a livebirth and a stillbirth) in our patients with PAI. Thirty-six percent of our pregnant patients had PAI. Only 1 of them needed long-term stress dosing during second trimester, and none developed adrenal crisis during delivery. The pregnancy complicated by development of PAI and adrenal crisis led to stillbirth at gestational week 24. Management of PAI in pregnancy can be challenging due to lack of evidence-based recommendations to guide glucocorticoid and mineralocorticoid dose adjustments (23). Moreover, diagnosis of new-onset adrenal insufficiency in pregnancy is challenging because symptoms are nonspecific and often not different from those commonly present in pregnancy itself, such as fatigue, nausea, and vomiting (30). In patients with APECED, positive 21-hydroxylase autoantibodies appear often before clinical symptoms of PAI. Patients with 21-hydroxylase autoantibodies should be monitored carefully for the development of PAI during pregnancy. With careful endocrine follow-up of women with adrenal insufficiency, maternal and fetal outcome is generally good (23).

The clinical spectrum of APECED disease manifestations remained mostly stable during pregnancy. Many autoimmune diseases tend to ameliorate during pregnancy (eg, Graves’ disease) (2), while others such as systemic lupus erythematosus may aggravate (31). Only 1 of our 43 patients developed a new APECED manifestation while pregnant, and alopecia worsened in 1 patient. Even though CMC is a common manifestation in APECED and genital candidiasis could exacerbate in changing hormonal milieu during pregnancy (32), no worsening of CMC symptoms was reported.

Pregnancy can also pose challenges for replacement therapy. There were altogether 46 pregnancies in our patients with HP, and in 5 pregnancies, moderate modifications of medications were required due to both hypocalcemia and hypercalcemia. During pregnancy, total serum calcium declines due to increased intravascular volume. However, as pregnancy progresses, increased production of parathyroid hormone-related protein with enhanced synthesis of 1,25(OH)_2_D_3_ may lead to increases in maternal blood calcium and reduced dose requirements for treatment of HP. Poorly controlled maternal hypocalcemia may lead to neonatal hyperparathyroidism while chronic maternal hypercalcemia may result in fetal secondary HP. Inadequate treatment of HP may result in uterine contractions and increased risk of miscarriage (33). A recent case series of HP during pregnancy reported an increased rate of caesarean delivery, polyhydramnios, and perinatal hypoxia (34). Another registry-based study found an increased proportion of induced labor and a slightly lower infant birth weight among 97 mothers with HP, but the overall risks were considered low (35). We did not observe increased rate of miscarriages or fetal complications due to hypo- or hypercalcemia.

We observed 2 stillbirths at gestational weeks 34 and 37; 1 was related to adrenal crisis. There was 1 neonatal death in a premature infant. No other serious perinatal complications were observed. This suggests that autoantibodies in patients with APECED do not cause significant autoimmune processes in the newborn babies. This is in contrast to autoimmune hyperthyroidism, a disease in which maternal activating thyroid-stimulating hormone receptor antibodies may lead to thyrotoxicosis in the baby (2) or autoantibodies in mothers with systemic lupus erythematosus may lead to bradycardia or neonatal lupus in the newborn (31).

Limitations of our study include possible missing data. There were certain differences in data collection; part of the information was collected from national registries, while part was based on self-reported material and medical records. We lacked information regarding how many women had tried but not succeeded in becoming pregnant. Women with severe phenotypes may not have tried to conceive. Due to the retrospective nature of the study, the patients had not been followed-up according to same protocols, and we were unable to include certain variables in the analyses (eg, the presence of 21-hydroxylase or side chain cleavage antibodies or levels of anti-Müllerian hormone at the time of POI diagnosis). Considering the multicentric nature of the study with data from different countries and across decades, a comparison of fertility with the respective general population was not possible. The strengths of our study include the availability of an extensive multicenter data set reflecting fetal and maternal outcome of pregnancies in women with APECED from a variety of countries.

Future studies are needed to elucidate the possible role of *AIRE* defects in fertility in humans. Because of the risk for early POI, the possibility of fertility preservation by prophylactic oocyte or ovarian tissue freezing should be considered. In our study, ODs were performed in only 2 countries, and the outcome of OD pregnancies was reassuring.

Management of APECED patients includes hormone and/or electrolyte replacement for irreversible endocrinopathies and immunosuppressive treatment to control autoimmunity in severe nonendocrine end-organ disease (11), Since all these therapies may have effects on fertility and pregnancy outcome of women with APECED, a multidisciplinary team is needed preconceptionally, during pregnancy, at delivery, and during puerperium. Close monitoring of hormone, mineral, and electrolyte replacement is important to control maternal calcium levels and to avoid acute adrenal or hypocalcemic crises.

In conclusion, the majority of women with APECED had normal pregnancy outcome, and the overall risks must be considered low. Many of the maternal APECED manifestations may, however, have a potentially negative impact on pregnancy prognosis warranting a careful multidisciplinary follow-up.

## Data Availability

Restrictions apply to the availability of some or all data generated or analyzed during this study to preserve patient confidentiality. The corresponding author will on request detail the restrictions and any conditions under which access to some data may be provided.

## Additional Information

**Disclosures:** The authors declare no competing interests.
